# Supplementary figures and images for: Infection-related severe maternal outcomes and case fatality rates in 43 low and middle-income countries across the WHO regions: Results from the Global Maternal Sepsis Study (GLOSS)
Source: PLOS Glob Public Health. 2024 Apr 25;4(4):e0003109. doi: 10.1371/journal.pgph.0003109 (PMC11045079; doi:10.1371/journal.pgph.0003109)

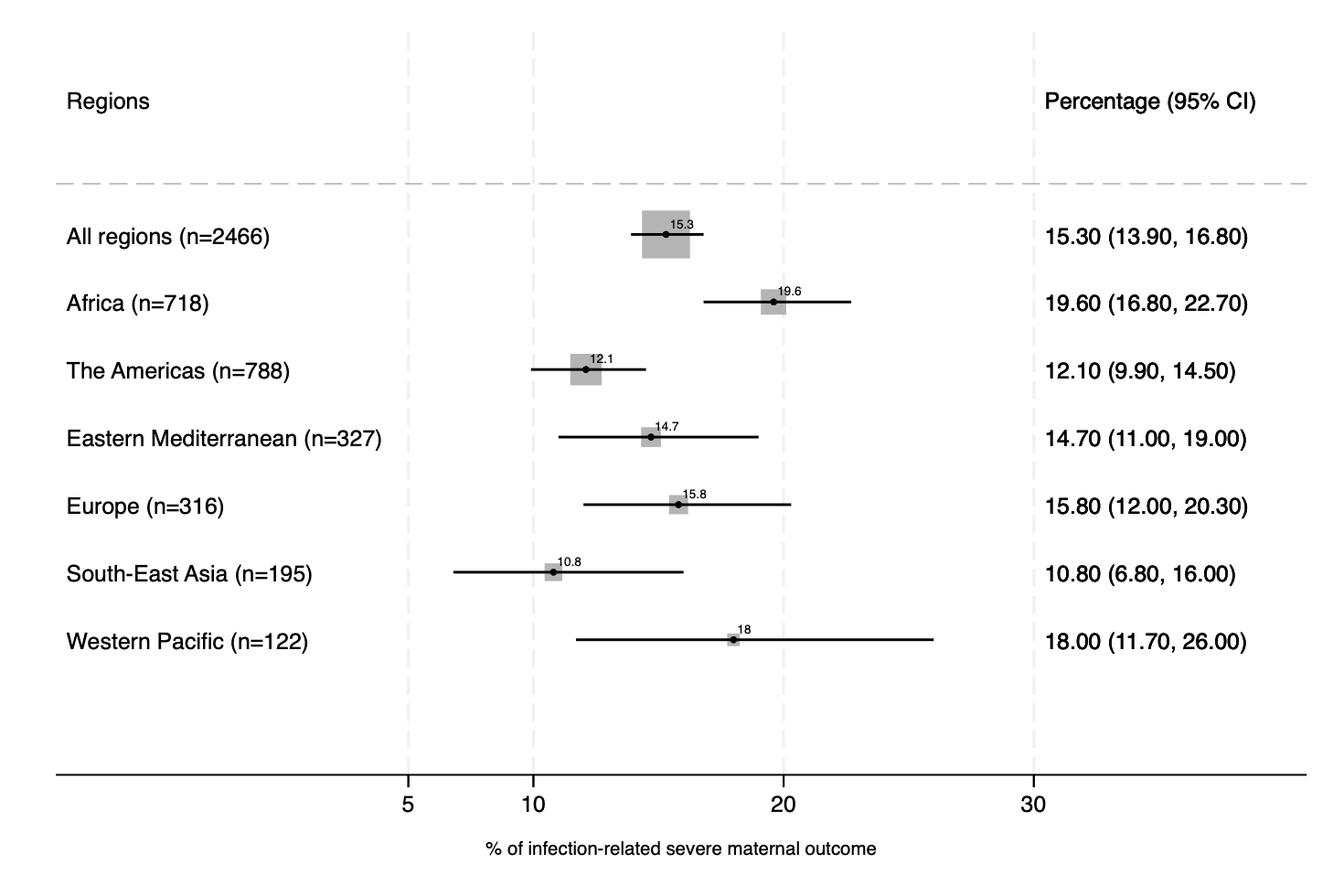

Supplement: S1 Fig — (TIF) [file pgph.0003109.s001.tif]
