# Supplementary material for: Infection-related severe maternal outcomes and case fatality rates in 43 low and middle-income countries across the WHO regions: Results from the Global Maternal Sepsis Study (GLOSS)
Source: PLOS Glob Public Health. 2024 Apr 25;4(4):e0003109. doi: 10.1371/journal.pgph.0003109 (PMC11045079; doi:10.1371/journal.pgph.0003109)
Supplement: S1 Table — (DOCX) [file pgph.0003109.s002.docx]

Appendix 1: Binary variables used to compute the composite variables.

| **Services available and functioning** | **Tests and procedures available** | **Treatment, drugs, and procedures available for pregnant and recently pregnant women** |
| --- | --- | --- |
| Adults' ICU | Temperature | Injectable ampicillin, gentamycin, or clindamycin |
| Adults' high dependency beds/unit | Blood pressure | Antimalaria drug |
| Neonatal intensive care unit | Oxygen saturation | Antiretroviral drugs |
| Newborn care unit with incubator | Urine dipstick for glucose, protein, ketone, and bodies | Uterotonics |
| Post-abortion services | Urinalysis | Magnesium sulfate |
| Blood Bank | Electrolytes | Antihypertensive agents |
| Blood donors screening for HIV, Hepatitis B and C, Syphilis | Glucose | Intravenous fluid |
| Biochemical/clinical laboratories | Creatinine | Blood products |
| Surgical theatre | Bilirubin | Manual removal of placenta |
| Ultrasound department | Lactate | Removal of retained products of conception |
| Radiology department | Liver function tests | Assisted vaginal delivery |
| **Resources in the facility:** | Hemoglobin | Caesarean section |
| Electricity | White blood cell count | Uninterrupted oxygen/CPAC |
| Generator | Platelets count | Mechanical ventilation |
| Incinerator | Coagulation test | Dialysis |
| Sewerage system | Blood group and cross-matching | Hysterectomy following infection or hemorrhage |
| Sterilization facility/equipment | Blood gas analysis/Gazometry | Use of continuous vasoactive drugs |
| Disinfectant for instruments | Lumbar punction | Cardio-pulmonary resuscitation |
| Landline or radio | Gram stains | Dialysis for acute renal failure |
| Ambulance | Blood culture | Any non-anesthetic intubation and  ventilation |
|  | Sputum culture | Transfusion of > 5 units of blood or red cells |
|  | ATB susceptibility test | **Availability of written protocols for:** |
|  | Malaria testing | Use of any checklists or job aids for essential childbirth care  (e.g., partograph) |
|  | HIV testing | Antibiotics for caesarean section |
|  | Syphilis testing | Restrictive episiotomy |
|  | **Clinical capacity to identify:** | Management of adult sepsis |
|  | Acute cyanosis | Management of newborn sepsis |
|  | Gasping | **Availability of written protocol for the use of prophylactic antibiotics for:** |
|  | Respiratory rate | Preterm labor with intact membranes |
|  | Shock | Preterm pre-labour rupture of membranes |
|  | Cardiac Arrest | Before or during a caesarean section |
|  | Oliguria | After manual removal of the placenta |
|  | Any loss of consciousness lasting | Third- or fourth-degree perineal tears |
|  | Stroke | Surgical abortion |
|  | Uncontrollable fit/status epilepticus | Neonate with risk factors for infection |
|  | Global paralysis |  |
|  | Jaundice in the presence of pre-eclampsia |  |
|  | Failure to form clots |  |
|  | **Laboratory capacity to identify:** |  |
|  | O2 saturation <90% for more than 60 min |  |
|  | PAO2/FiO2<200 mmHg |  |
|  | Creatinine>300 umol/ml or > 3.5mg/dL |  |
|  | Bilirubin>100umol/L or >6.0mg/dL |  |
|  | pH<7.121 |  |
|  | Lactate > 5mEq/L |  |

Note: each variable is coded 1 if mentioned and 0 if not mentioned
