# Supplementary material for: Infection-related severe maternal outcomes and case fatality rates in 43 low and middle-income countries across the WHO regions: Results from the Global Maternal Sepsis Study (GLOSS)
Source: PLOS Glob Public Health. 2024 Apr 25;4(4):e0003109. doi: 10.1371/journal.pgph.0003109 (PMC11045079; doi:10.1371/journal.pgph.0003109)
Supplement: S2 Table — (DOCX) [file pgph.0003109.s003.docx]

Appendix 1: Services and equipment available in the facility

| Services and equipment | Africa | | The Americas | | Eastern Mediterranean | | Europe | | South-East Asia | | Western Pacific | | ALL | |
| --- | --- | --- | --- | --- | --- | --- | --- | --- | --- | --- | --- | --- | --- | --- |
| **Services available and functioning on the day of the visit** | n | % | n | % | n | % | n | % | n | % | n | % | N | % |
| Adults' ICU | 64 | 50.8 | 74 | 84.1 | 28 | 60.9 | 53 | 93.0 | 18 | 51.4 | 50 | 89.3 | 287 | 70.3 |
| Adults' high dependency beds/unit | 59 | 46.8 | 70 | 79.5 | 34 | 73.9 | 40 | 70.2 | 19 | 54.3 | 25 | 45.5 | 247 | 60.7 |
| Neonatal intensive care unit | 62 | 49.2 | 76 | 86.4 | 25 | 54.3 | 36 | 63.2 | 19 | 54.3 | 37 | 66.1 | 255 | 62.5 |
| Other newborn care unit with incubator | 72 | 57.1 | 79 | 89.8 | 30 | 65.2 | 39 | 68.4 | 26 | 74.3 | 41 | 73.2 | 287 | 70.3 |
| Abortion services | 110 | 87.3 | 82 | 93.2 | 43 | 93.5 | 53 | 96.4 | 26 | 74.3 | 46 | 82.1 | 360 | 88.7 |
| Post-abortion services | 115 | 91.3 | 83 | 94.3 | 44 | 95.6 | 55 | 96.5 | 34 | 97.1 | 43 | 76.8 | 374 | 91.7 |
| Blood Bank | 77 | 61.1 | 81 | 93.1 | 30 | 65.2 | 39 | 68.4 | 26 | 74.3 | 28 | 50.0 | 281 | 69.0 |
| Blood donors screening for HIV, Hepatitis B and C, Syphilis | 101 | 80.2 | 87 | 98.9 | 31 | 67.4 | 34 | 60.7 | 32 | 91.4 | 41 | 73.2 | 326 | 80.1 |
| Biochemical/clinical laboratories | 119 | 94.4 | 87 | 98.9 | 42 | 91.3 | 54 | 94.7 | 35 | 100.0 | 47 | 83.9 | 384 | 94.1 |
| Surgical theatre | 108 | 85.7 | 86 | 97.7 | 46 | 100.0 | 56 | 98.3 | 27 | 77.1 | 53 | 94.6 | 376 | 92.2 |
| Ultrasound department | 106 | 84.1 | 87 | 98.9 | 42 | 91.3 | 54 | 94.7 | 31 | 88.6 | 53 | 94.6 | 373 | 91.4 |
| Radiology department | 102 | 80.9 | 83 | 94.3 | 45 | 97.8 | 38 | 66.7 | 30 | 85.7 | 52 | 92.9 | 350 | 85.8 |
| On-site pharmacy | 109 | 96.5 | 41 | 97.6 | 42 | 95.4 | 36 | 85.7 | 32 | 91.4 | 28 | 90.3 | 288 | 93.8 |
| **Resources in the facility** |  |  |  |  |  |  |  |  |  |  |  |  |  |  |
| *Electricity* | 122 | 96.8 | 88 | 100.0 | 46 | 100.0 | 57 | 100.0 | 33 | 94.3 | 56 | 100.0 | 402 | 98.5 |
| *Generator* | 116 | 92.1 | 85 | 96.6 | 46 | 100.0 | 55 | 96.5 | 33 | 94.3 | 56 | 100.0 | 391 | 95.8 |
| *Incinerator* | 92 | 73.0 | 44 | 50.0 | 28 | 60.9 | 32 | 56.1 | 23 | 65.7 | 42 | 75.0 | 261 | 64.0 |
| *Sewerage system* | 101 | 82.1 | 87 | 98.9 | 46 | 100.0 | 56 | 98.2 | 35 | 100.0 | 50 | 89.3 | 375 | 92.6 |
| *Sterilization facility/equipment* | 123 | 97.6 | 86 | 97.7 | 46 | 100.0 | 57 | 100.0 | 35 | 100.0 | 55 | 98.2 | 402 | 98.5 |
| *Disenfectant for instruments* | 121 | 96.0 | 88 | 100.0 | 46 | 100.0 | 57 | 100.0 | 35 | 100.0 | 55 | 98.2 | 402 | 98.5 |
| *Landline or radio* | 100 | 79.4 | 88 | 100.0 | 40 | 87.0 | 54 | 100.0 | 35 | 100.0 | 55 | 98.2 | 372 | 91.8 |
| Ambulance | 93 | 73.8 | 73 | 82.9 | 38 | 82.6 | 51 | 89.5 | 33 | 94.3 | 56 | 100.0 | 344 | 84.3 |
