# Supplementary material for: Infection-related severe maternal outcomes and case fatality rates in 43 low and middle-income countries across the WHO regions: Results from the Global Maternal Sepsis Study (GLOSS)
Source: PLOS Glob Public Health. 2024 Apr 25;4(4):e0003109. doi: 10.1371/journal.pgph.0003109 (PMC11045079; doi:10.1371/journal.pgph.0003109)
Supplement: S3 Table — (DOCX) [file pgph.0003109.s004.docx]

Appendix 3: Identification and diagnostic capacity

|  | **Africa** | | **The Americas** | | **Eastern Mediterranean** | | **Europe** | | **South-East Asia** | | **Western Pacific** | | **ALL** | |
| --- | --- | --- | --- | --- | --- | --- | --- | --- | --- | --- | --- | --- | --- | --- |
| **Tests available on the day of the visit for** | n | % | n | % | n | % | n | % | n | % | n | % | N | % |
| Temperature | 126 | 100.0 | 88 | 100.0 | 46 | 100.0 | 55 | 100.0 | 35 | 100.0 | 52 | 92.9 | 402 | 99.0 |
| Blood pressure | 126 | 100.0 | 88 | 100.0 | 46 | 100.0 | 57 | 100.0 | 35 | 100.0 | 52 | 92.9 | 404 | 99.0 |
| Oxygen saturation | 94 | 74.6 | 88 | 100.0 | 43 | 93.5 | 57 | 100.0 | 33 | 94.3 | 48 | 85.7 | 363 | 89.0 |
| Urine dipstick for glucose, protein, ketone, and bodies | 125 | 99.2 | 86 | 97.7 | 44 | 95.7 | 38 | 66.7 | 34 | 97.1 | 49 | 87.5 | 376 | 92.2 |
| Urinalysis | 108 | 85.7 | 88 | 100.0 | 43 | 93.5 | 55 | 100.0 | 35 | 100.0 | 43 | 91.5 | 372 | 93.7 |
| Electrolytes | 108 | 86.4 | 85 | 96.6 | 40 | 87.0 | 54 | 94.7 | 32 | 91.4 | 49 | 87.5 | 368 | 90.4 |
| Glucose | 119 | 94.4 | 87 | 98.9 | 43 | 93.5 | 57 | 100.0 | 35 | 100.0 | 55 | 98.2 | 396 | 97.1 |
| Creatinine | 107 | 84.9 | 87 | 98.9 | 44 | 95.6 | 57 | 100.0 | 33 | 94.3 | 52 | 92.9 | 380 | 93.1 |
| Bilirubin | 94 | 74.6 | 86 | 97.7 | 42 | 91.3 | 56 | 98.2 | 33 | 94.3 | 52 | 92.9 | 363 | 89.0 |
| Lactate | 62 | 49.2 | 84 | 95.4 | 20 | 43.5 | 47 | 82.5 | 24 | 68.6 | 36 | 64.3 | 273 | 66.9 |
| Liver function tests | 104 | 82.5 | 87 | 98.9 | 42 | 91.3 | 57 | 100.0 | 31 | 88.6 | 53 | 94.6 | 374 | 91.7 |
| Hemoglobin | 118 | 93.6 | 88 | 100.0 | 44 | 95.6 | 56 | 98.3 | 35 | 100.0 | 52 | 92.9 | 393 | 96.3 |
| White blood cell count | 115 | 91.3 | 86 | 97.7 | 44 | 95.6 | 55 | 100.0 | 33 | 94.3 | 53 | 94.6 | 386 | 95.1 |
| Platelets count | 115 | 91.3 | 87 | 98.9 | 44 | 95.6 | 57 | 100.0 | 34 | 97.1 | 53 | 94.6 | 390 | 95.6 |
| Coagulation test | 80 | 63.5 | 85 | 96.6 | 40 | 87.0 | 50 | 87.7 | 31 | 88.6 | 50 | 89.3 | 336 | 82.3 |
| Blood group and cross-matching | 114 | 90.5 | 88 | 100.0 | 41 | 89.1 | 55 | 96.5 | 35 | 100.0 | 55 | 98.2 | 388 | 95.1 |
| Blood gas analysis/Gazometry | 45 | 35.7 | 84 | 95.4 | 19 | 41.3 | 48 | 84.2 | 16 | 45.7 | 16 | 28.6 | 228 | 55.9 |
| Lumbar punction | 108 | 85.7 | 83 | 95.4 | 32 | 69.6 | 49 | 89.1 | 25 | 71.4 | 40 | 71.4 | 337 | 83.2 |
| Gram stains | 103 | 81.7 | 86 | 97.7 | 26 | 56.5 | 34 | 61.8 | 31 | 88.6 | 41 | 73.2 | 321 | 79.1 |
| Blood culture | 64 | 50.8 | 84 | 95.4 | 26 | 56.5 | 36 | 63.2 | 25 | 71.4 | 25 | 44.6 | 260 | 63.7 |
| Sputum culture | 77 | 61.1 | 83 | 94.3 | 27 | 58.7 | 38 | 66.7 | 26 | 74.3 | 29 | 51.8 | 280 | 68.6 |
| Antibiotics susceptibility test | 79 | 62.7 | 86 | 97.7 | 29 | 63.0 | 46 | 83.6 | 24 | 68.6 | 50 | 90.9 | 314 | 77.5 |
| Malaria testing | 124 | 98.4 | 69 | 79.3 | 24 | 52.2 | 14 | 24.6 | 31 | 88.6 | 32 | 57.1 | 294 | 72.2 |
| HIV testing | 126 | 100.0 | 86 | 97.7 | 37 | 80.4 | 54 | 94.7 | 35 | 100.0 | 55 | 98.2 | 393 | 96.3 |
| Syphilis testing | 121 | 96.0 | 86 | 97.7 | 34 | 73.9 | 50 | 87.7 | 34 | 97.1 | 34 | 61.8 | 359 | 88.2 |
| **Clinical capacity to identify:** |  |  |  |  |  |  |  |  |  |  |  |  |  |  |
| Acute cyanosis | 117 | 92.9 | 83 | 95.4 | 46 | 100.0 | 56 | 100.0 | 34 | 97.1 | 49 | 87.5 | 385 | 94.8 |
| Gasping | 114 | 91.2 | 84 | 96.6 | 45 | 97.8 | 56 | 100.0 | 34 | 97.1 | 47 | 83.9 | 380 | 93.8 |
| Respiratory rate>40 or <6 bpm | 115 | 92.7 | 85 | 96.6 | 45 | 97.8 | 56 | 100.0 | 35 | 100.0 | 47 | 83.9 | 383 | 94.6 |
| Shock | 123 | 97.6 | 87 | 98.9 | 46 | 100.0 | 56 | 100.0 | 35 | 100.0 | 50 | 89.3 | 397 | 97.5 |
| Cardiac Arrest | 115 | 91.3 | 84 | 96.6 | 46 | 100.0 | 56 | 100.0 | 33 | 94.3 | 46 | 82.1 | 380 | 93.6 |
| Oliguria non-responsive to fluids or diuretics | 118 | 93.7 | 87 | 98.9 | 43 | 93.5 | 56 | 100.0 | 33 | 94.3 | 47 | 83.9 | 384 | 94.3 |
| Any loss of consciousness lasting | 119 | 94.4 | 84 | 96.6 | 45 | 100.0 | 55 | 98.2 | 33 | 94.3 | 40 | 71.4 | 376 | 92.8 |
| Stroke | 113 | 89.7 | 81 | 93.1 | 43 | 93.5 | 54 | 96.4 | 32 | 91.4 | 41 | 73.2 | 364 | 89.7 |
| Uncontrollable fit/status epilepticus | 120 | 95.2 | 80 | 91.9 | 44 | 95.7 | 55 | 98.2 | 34 | 97.1 | 46 | 82.1 | 379 | 93.3 |
| Global paralysis | 112 | 88.9 | 78 | 89.7 | 38 | 82.6 | 53 | 94.6 | 31 | 88.6 | 39 | 69.6 | 351 | 86.4 |
| Jaundice in the presence of pre-eclampsia | 117 | 93.6 | 81 | 92.1 | 45 | 97.8 | 53 | 94.6 | 32 | 91.4 | 42 | 75.0 | 370 | 91.1 |
| Failure to form clots | 112 | 89.6 | 84 | 95.5 | 43 | 93.5 | 55 | 98.2 | 31 | 88.6 | 38 | 67.9 | 363 | 89.4 |
| **Laboratory capacity for identification of:** |  |  |  |  |  |  |  |  |  |  |  |  |  |  |
| O2 saturation <90% for more than 60 min | 81 | 64.3 | 83 | 95.4 | 35 | 76.1 | 55 | 96.5 | 32 | 91.4 | 43 | 78.2 | 329 | 81.0 |
| PAO2/FiO2<200 mmHg | 45 | 35.7 | 83 | 95.4 | 26 | 56.5 | 53 | 93.0 | 19 | 54.3 | 27 | 49.1 | 253 | 62.3 |
| Creatinine>300 umol/ml or > 3.5mg/dL | 98 | 77.8 | 83 | 95.4 | 40 | 87.0 | 55 | 96.5 | 34 | 97.1 | 48 | 88.9 | 358 | 88.4 |
| Bilirubin>100umol/L or >6.0mg/dL | 94 | 74.6 | 84 | 96.6 | 39 | 84.8 | 55 | 96.5 | 34 | 97.1 | 46 | 85.2 | 352 | 86.9 |
| pH<7.121 | 51 | 40.5 | 82 | 94.3 | 22 | 47.8 | 53 | 93.0 | 23 | 65.7 | 33 | 60.0 | 264 | 65.0 |
| Lactate > 5mEq/L | 34 | 29.8 | 82 | 94.3 | 12 | 28.6 | 48 | 92.3 | 20 | 57.1 | 27 | 50.0 | 223 | 58.1 |
