# Supplementary material for: Infection-related severe maternal outcomes and case fatality rates in 43 low and middle-income countries across the WHO regions: Results from the Global Maternal Sepsis Study (GLOSS)
Source: PLOS Glob Public Health. 2024 Apr 25;4(4):e0003109. doi: 10.1371/journal.pgph.0003109 (PMC11045079; doi:10.1371/journal.pgph.0003109)
Supplement: S4 Table — (DOCX) [file pgph.0003109.s005.docx]

Appendix 4: Infection management-related characteristics of the facilities by region

|  | Africa | | The Americas | | Eastern Mediterranean | | Europe | | South-East Asia | | Western Pacific | | ALL | |
| --- | --- | --- | --- | --- | --- | --- | --- | --- | --- | --- | --- | --- | --- | --- |
| **Treatment/medicine available for pregnant and recently pregnant women** | n | % | n | % | n | % | n | % | n | % | n | % | N | % |
| Any injectable antibiotic | 125 | 99.2 | 83 | 95.4 | 46 | 100.0 | 57 | 100.0 | 35 | 100.0 | 53 | 94.6 | 399 | 98.0 |
| Injectable ampicillin, gentamycin or clindamycin | 126 | 100.0 | 88 | 100.0 | 45 | 97.8 | 54 | 98.2 | 34 | 97.1 | 56 | 100.0 | 403 | 99.3 |
| Antimalaria drug | 122 | 96.8 | 59 | 67.1 | 17 | 37.0 | 12 | 21.4 | 28 | 80.0 | 24 | 42.9 | 262 | 64.4 |
| Antiretroviral drugs | 120 | 95.2 | 76 | 86.4 | 15 | 32.6 | 32 | 56.1 | 32 | 91.4 | 30 | 53.6 | 305 | 74.7 |
| Uterotoniccs | 123 | 97.6 | 87 | 98.9 | 45 | 97.8 | 57 | 100.0 | 35 | 100.0 | 56 | 100.0 | 403 | 98.8 |
| Magnesium sulfate | 116 | 92.1 | 87 | 98.9 | 45 | 97.8 | 46 | 80.7 | 35 | 100.0 | 53 | 94.6 | 382 | 93.6 |
| Antihypertensive agents | 124 | 98.4 | 87 | 98.9 | 45 | 97.8 | 57 | 100.0 | 35 | 100.0 | 55 | 98.2 | 403 | 98.8 |
| Intravenous fluid | 122 | 96.8 | 87 | 98.9 | 44 | 95.6 | 57 | 100.0 | 33 | 94.3 | 56 | 100.0 | 399 | 97.8 |
| Blood products | 97 | 77.6 | 85 | 96.6 | 37 | 80.4 | 57 | 100.0 | 33 | 94.3 | 43 | 76.8 | 352 | 86.5 |
| Manual removal of placenta | 124 | 98.4 | 86 | 97.7 | 45 | 97.8 | 55 | 96.5 | 34 | 97.1 | 54 | 96.4 | 398 | 97.5 |
| Removal of retained products of conception | 121 | 96.0 | 85 | 96.6 | 45 | 97.8 | 55 | 96.5 | 35 | 100.0 | 53 | 94.6 | 394 | 96.6 |
| Assisted vaginal delivery | 102 | 80.9 | 87 | 98.9 | 45 | 97.8 | 52 | 91.2 | 34 | 97.1 | 52 | 92.9 | 372 | 91.2 |
| Caesarean section | 112 | 88.9 | 85 | 96.6 | 45 | 97.8 | 55 | 96.5 | 29 | 82.9 | 56 | 100.0 | 382 | 93.6 |
| Uninterrupted oxygen/CPAC | 101 | 80.2 | 85 | 96.6 | 41 | 89.1 | 55 | 96.5 | 27 | 77.1 | 45 | 80.4 | 354 | 86.8 |
| Mechanical ventilation | 94 | 74.6 | 81 | 92.0 | 43 | 93.5 | 54 | 94.7 | 25 | 71.4 | 43 | 76.8 | 340 | 83.3 |
| Dialysis | 33 | 26.2 | 55 | 63.2 | 25 | 54.3 | 14 | 24.6 | 20 | 57.1 | 16 | 28.6 | 163 | 40.0 |
| **Management indicators of severity** |  |  |  |  |  |  |  |  |  |  |  |  |  |  |
| Hysterectomy following infection or haemorrhage | 109 | 86.5 | 84 | 96.6 | 46 | 100.0 | 55 | 96.5 | 23 | 65.7 | 52 | 92.9 | 369 | 90.7 |
| Use of continuous vasoactive drugs | 97 | 77.0 | 84 | 95.5 | 41 | 89.1 | 56 | 98.3 | 31 | 88.6 | 53 | 94.6 | 362 | 88.7 |
| Cardio-pulmonary resuscitation | 105 | 83.3 | 87 | 98.9 | 39 | 84.8 | 55 | 96.5 | 34 | 97.1 | 54 | 96.4 | 374 | 91.7 |
| Dialysis for acute renal failure | 37 | 29.4 | 72 | 81.8 | 30 | 65.2 | 17 | 29.8 | 15 | 42.9 | 23 | 41.1 | 194 | 47.5 |
| Any non-anaesthetic intubation and  ventilation | 73 | 57.9 | 85 | 96.6 | 37 | 80.4 | 53 | 93.0 | 27 | 77.1 | 54 | 96.4 | 329 | 80.6 |
| Transfusion of > 5 units of blood or red cells | 84 | 66.7 | 84 | 95.5 | 38 | 82.6 | 53 | 93.0 | 23 | 65.7 | 45 | 83.3 | 327 | 80.5 |
| **Availability of written protocols** |  |  |  |  |  |  |  |  |  |  |  |  |  |  |
| Use of any checklists or job aids for essential childbirth care  (e.g., partograph, Safe Childbirth Checklist) | 103 | 81.8 | 78 | 89.7 | 42 | 91.3 | 52 | 96.3 | 28 | 80.0 | 47 | 83.9 | 350 | 86.6 |
| Antibiotics for caesarean section | 98 | 77.8 | 73 | 82.9 | 42 | 91.3 | 54 | 94.7 | 23 | 65.7 | 52 | 92.9 | 342 | 83.8 |
| Restrictive episiotomy | 85 | 67.5 | 68 | 78.2 | 35 | 76.1 | 30 | 52.6 | 26 | 74.3 | 51 | 91.1 | 295 | 72.5 |
| Management of adult sepsis | 86 | 68.2 | 71 | 80.7 | 34 | 73.9 | 51 | 89.5 | 28 | 80.0 | 49 | 87.5 | 319 | 78.2 |
| Management of newborn sepsis | 86 | 68.2 | 76 | 86.4 | 31 | 67.4 | 52 | 94.6 | 26 | 74.3 | 45 | 80.4 | 316 | 77.8 |
| **Do you have written protocols for the use of prophylactic antibiotics for :** |  |  |  |  |  |  |  |  |  |  |  |  |  |  |
| Preterm labor with intact membranes | 71 | 56.3 | 69 | 78.4 | 28 | 60.9 | 54 | 94.7 | 25 | 71.4 | 40 | 71.4 | 287 | 70.3 |
| Preterm prelabour rupture of membranes | 92 | 73.0 | 73 | 82.9 | 33 | 71.7 | 55 | 96.5 | 27 | 77.1 | 49 | 87.5 | 329 | 80.6 |
| Before or during a caesarean section | 85 | 68.0 | 71 | 80.7 | 34 | 73.9 | 53 | 93.0 | 21 | 60.0 | 51 | 91.1 | 315 | 77.4 |
| After manual removal of the placenta | 93 | 73.8 | 56 | 63.6 | 34 | 73.9 | 31 | 54.4 | 26 | 74.3 | 48 | 85.7 | 288 | 70.6 |
| Third- or fourth-degree perineal tears | 84 | 66.7 | 60 | 68.2 | 31 | 67.4 | 28 | 49.1 | 27 | 77.1 | 51 | 91.1 | 281 | 68.9 |
| Surgical abortion | 71 | 57.7 | 65 | 73.9 | 31 | 67.4 | 32 | 56.1 | 19 | 54.3 | 47 | 83.9 | 265 | 65.4 |
| Neonate with risk factors for infection | 85 | 67.5 | 72 | 81.8 | 30 | 65.2 | 47 | 100.0 | 25 | 71.4 | 47 | 83.9 | 306 | 76.9 |
