# Supplementary material for: Infection-related severe maternal outcomes and case fatality rates in 43 low and middle-income countries across the WHO regions: Results from the Global Maternal Sepsis Study (GLOSS)
Source: PLOS Glob Public Health. 2024 Apr 25;4(4):e0003109. doi: 10.1371/journal.pgph.0003109 (PMC11045079; doi:10.1371/journal.pgph.0003109)
Supplement: S1 Acknowledgments — (DOCX) [file pgph.0003109.s006.docx]

**WHO Global Maternal Sepsis Study Research Group members**:

**Afghanistan:** Mohammad Iqbal Aman, Bashir Noormal.

**Argentina:** Virginia Díaz, Marisa Espinoza, Julia Pasquale.

**Belgium:** Charlotte Leroy, Kristien Roelens, Griet Vandenberghe.

**Benin:** M Christian Urlyss Agossou, Sourou Goufodji Keke, Christiane Tshabu Aguemon.

**Bolivia:** Patricia Soledad Apaza Peralta, Víctor Conde Altamirano, Rosalinda Hernández Muñoz.

**Brazil:** José Guilherme Cecatti, Carolina C Ribeiro-Do-Valle.

**Burkina Faso:** Vincent Batiene, Kadari Cisse, Henri Gautier Ouedraogo.

**Cambodia:** Cheang Kannitha, Lam Phirun, Tung Rathavy.

**Cameroon:** Elie Simo, Pierre-Marie Tebeu, Emah Irene Yakana.

**Colombia:** Javier Carvajal, María Fernanda Escobar, Paula Fernández.

**Denmark:** Lotte Berdiin Colmorn, Jens Langhoff-Roos.

**Ecuador:** Wilson Mereci, Paola Vélez.

**Egypt:** Yasser Salah Eldin, Alaa Sultan.

**Ethiopia:** Abdulfetah Abdulkadir Abdosh, Alula M Teklu, Dawit Worku Kassa.

**Ghana:** Richard Adanu, Philip Govule, Charles Noora Lwanga.

**Guatemala:** William Enrique Arriaga Romero, María Guadalupe Flores Aceituno.

**Honduras:** Carolina Bustillo, Rigoberto Castro, Bredy Lara.

**India:** Vijay Kumar, Vanita Suri, Sonia Trikha.

**Italy:** Irene Cetin, Serena Donati, Carlo Personeni.

**Kazakhstan:** Guldana Baimussanova, Saule Kabylova, Balgyn Sagyndykova.

**Kenya:** George Gwako, Alfred Osoti, Zahida Qureshi.

**Kyrgyzstan:** Raisa Asylbasheva, Aigul Boobekova, Damira Seksenbaeva.

**Lebanon:** Faysal El Kak, Saad Eddine Itani, Sabina Abou Malham.

**Lithuania:** Meilė Minkauskienė, Diana Ramašauskaitė.

**Malawi:** Owen Chikhwaza, Luis Gadama, Eddie Malunga.

**Mali:** Haoua Dembele, Hamadoun Sangho, Fanta Eliane Zerbo.

**Mexico:** Filiberto Dávila Serapio, Nazarea Herrera Maldonado, Juan Ismael Islas Castañeda.

**Moldova:** Tatiana Caraus, Ala Curteanu, Victor Petrov.

**Mongolia:** Yadamsuren Buyanjargal, Seded Khishgee, Bat-Erdene Lkhagvasuren.

**Morocco:** Bouchra Assarag, Amina Essolbi, Rachid Moulki.

**Mozambique:** Nafissa Bique Osman, Zara Jaze, Arlete Mariano.

**Myanmar:** Hla Mya Thway Einda, Thae Maung Maung, Khaing Nwe Tin.

**Nepal:** Tara Gurung, Amir Babu Shrestha, Sangeeta Shrestha.

**Netherlands:** Kitty Bloemenkamp, Marcus J Rijken, Thomas Van Den Akker.

**Nicaragua:** María Esther Estrada, Néstor J Pavón Gómez.

**Nigeria:** Olubukola Adesina, Chris Aimakhu, Bukola Fawole.

**Pakistan:** Rizwana Chaudhri, Saima Hamid, M Adnan Khan.

**Peru:** María del Pilar Huatuco Hernández, Nelly M Zavaleta Pimentel.

**Philippines:** Maria Lu Andal, Carolina Paula Martin, Zenaida Dy Recidoro.

**Romania:** Mihaela-Alexandra Budianu, Lucian Pușcașiu.

**Senegal:** Léopold Diouf, Dembo Guirassy, Philippe Marc Moreira.

**Slovakia:** Miroslav Borovsky, Ladislav Kovac, Alexandra Kristufkova.

**South Africa:** Sylvia Cebekhulu, Laura Cornelissen, Priya Soma-Pillay.

**Spain:** Vicenç Cararach, Marta López, María José Vidal Benedé.

**Sri Lanka:** Hemali Jayakody, Kapila Jayaratne, Dhammica Rowel.

**Sudan:** Mohamed Elsheikh, Wisal Nabag, Sara Omer.

**Tajikistan:** Victoria Tsoy, Urunbish Uzakova, Dilrabo Yunusova.

**Thailand:** Thitiporn Siriwachirachai, Thumwadee Tangsiriwatthana.

**UK:** Catherine Dunlop, Marian Knight, David Lissauer.

**Uruguay:** Aquilino M Pérez, Jhon Roman, Gerardo Vitureira.

**Viet Nam:** Dinh Anh Tuan, Luong Ngoc Truong, Nghiem Thi Xuan Hanh.

**Zimbabwe:** Mugove Madziyire, Thulani Magwali, Stephen Munjanja.

**Regional Coordinators:** Edgardo Abalos, Adama Baguiya, Mónica Chamillard, Bukola Fawole, Marian Knight, Seni Kouanda, Pisake Lumbiganon, Ashraf Nabhan, Ruta Nadisauskiene.

**Technical Advisory Group:** Linda Bartlett, Fernando Bellissimo-Rodrigues, Shevin T Jacob, Sadia Shakoor, Khalid Yunis.

**Data management and analysis:** Liana Campodónico, Cristina Cuesta, Hugo Gamerro, Daniel Giordano.

**WHO coordinating Unit:** Fernando Althabe, Mercedes Bonet, Vanessa Brizuela, A Metin Gülmezoglu, João Paulo Souza.
