# Supplementary material for: Infection-related severe maternal outcomes and case fatality rates in 43 low and middle-income countries across the WHO regions: Results from the Global Maternal Sepsis Study (GLOSS)
Source: PLOS Glob Public Health. 2024 Apr 25;4(4):e0003109. doi: 10.1371/journal.pgph.0003109 (PMC11045079; doi:10.1371/journal.pgph.0003109)
Supplement: Attachment — Submitted filename: Response to reviewers_vf.docx [file pgph.0003109.s007.docx]

Infection-related severe maternal outcomes and case fatality rates in 43 Low and Middle-Income Countries across the WHO regions: results from the Global Maternal Sepsis Study (GLOSS)

Dear Editor,

We would like to thank you for your assistance and comments on our manuscript entitled: “Infection-related severe maternal outcomes and case fatality rates in 43 Low and Middle-Income Countries across the WHO regions: results from the Global Maternal Sepsis Study (GLOSS).” We benefited from very relevant comments from you and the reviewers. In the current version, we addressed all the comments and hope our responses and actions taken to improve the manuscript will meet your expectations. We are fully available to consider any additional comment or suggestion that you or the reviewers will have on the current version as well.

Please find below a point by point list of the comments and how we addressed them

Comments of the Editor

1. In the online submission form, you indicated that "The data used for this analysis can be made available upon request. Contact of the GLOSS coordinator [bonetm@who.int](mailto:bonetm@who.int)."

 All PLOS journals now require all data underlying the findings described in their manuscript to be freely available to other researchers, either 
1. In a public repository, 
2. Within the manuscript itself, or 
3. Uploaded as supplementary information.

This policy applies to all data except where public deposition would breach compliance with the protocol approved by your research ethics board. If your data cannot be made publicly available for ethical or legal reasons (e.g., public availability would compromise patient privacy), please explain your reasons by return email and your exemption request will be escalated to the editor for approval. Your exemption request will be handled independently and will not hold up the peer review process, but will need to be resolved should your manuscript be accepted for publication. One of the Editorial team will then be in touch if there are any issues.

Response

Following the WHO policy for sharing and reuse of research data, the analytic datasets containing individual participant level data from this multi-country study data are made available under data share agreement between the requester or requesting institution and the study institutions. A request for these data may be made to the editorial board by emailing to (**enter contact-info here**) with the concept paper and contact information.

While we fully understand and support the data transparency and public availability policy in relation to data sharing, we will request an exemption because the agreement with the countries’ teams was that the countries own their dataset, and together with the global study coordination, own the global data. The country-specific datasets were returned to them, and the WHO archived the full dataset. Any request for global data has to go to the study coordination, and those on country data are submitted to that country. All the collaboration was based on this agreement. That is why each and every country obtained approval from their government and submitted to ethics in their own country, and the coordination submitted to the WHO ERC. Therefore, while we fully support the data-sharing policy, releasing the dataset in a public repository is against our memorandum of understanding between the study entities. We will consider including this policy in our future collaboration for global studies.

2. Please review your reference list to ensure that it is complete and correct. If you have cited papers that have been retracted, please include the rationale for doing so in the manuscript text, or remove these references and replace them with relevant current references. Any changes to the reference list should be mentioned in the rebuttal letter that accompanies your revised manuscript. If you need to cite a retracted article, indicate the article’s retracted status in the References list and also include a citation and full reference for the retraction notice.

Response

References 1 and 31 have been removed. Reference 1 was removed from its text to shorten the introduction. Reference 31 was referring to maternal mortality in 2017. It was updated by the report for 2020, which was already cited in the manuscript.

Responses to reviewer’s comments

Please use the space provided to explain your answers to the questions above. You may also include additional comments for the author, including concerns about dual publication, research ethics, or publication ethics. (Please upload your review as an attachment if it exceeds 20,000 characters)

Reviewer #1:

I read the article with big interest and I have nothing to add. The article is written very clearly, methodology is well described, results are completely illustrated in tables and figures, limits of the study are explained and conclusions are clear.

R: Thank you for your appreciation

Reviewer #2: Dear authors and co-authors, thank you for reviewing an interesting manuscript.
Please see the specified questions and suggestions below.

1. Complete manuscript:
The manuscript should be checked by an English native speaker, there are unnecessary errors obscuring the quality of the study.

Response:

The manuscript has been now checked by on of the authors who is English native speaker.

Abstract:
2. Lines 31-34: "The highest toll of maternal mortality due to infections is reported in low- and middle-income countries (LMICs). However, more evidence is needed to understand the differences related to severe maternal outcomes (SMO) and fatality rates across the WHO regions."
I would suggest adding the term “due to infections” to clarify that the study only concerns SMO and fatality rates due to infections.

Response: We revised the paragraph accordingly and it now reads: *“The highest toll of maternal mortality due to infections is reported in low- and middle-income countries (LMICs). However, more evidence is needed to understand the differences in infection-related severe maternal outcomes (SMO) and fatality rates across the WHO regions.”*

4. Line 40: "SMOs were infection-related maternal deaths or near-miss."
Should be changed to "SMOs are defined as maternal deaths and maternal near-misses."

Response: thank you for the suggestion. We made this change.

5. Sentences 41-42: The meaning is not clear, what does “on a set of countries and facilities characteristics” mean? Please clarify.

Response: We wanted to indicate that the odd ratios were adjusted on a set of countries and facilities characteristics. But we agree with the reviewer that this was poorly stated and therefore we removed the statement between brackets.

6. Lines 44-51: The results seem haphazardly presented, as a reader I would expect to find summarized results (Africa versus the other regions combined for example). Please consider revising.

Response:

While we understand the reviewer's concern, we believe that combining all the other regions together may lead to confusion with pooled information that is not geographically meaningful. We therefore added the proportions of the other regions one by one to make the comparison with AFRO.

Secondly, the aOR are from the comparison of AFRO versus the other regions one by one.

7. Lines 62-64: I would expect some kind of comparison to be able to judge the percentage incidence of direct obstetric infection in sub-Saharan Africa. Standing alone, it is difficult to judge.

Response

We added the figures from North America and Western Europe as reported by Sakr et al 2018 for comparison purpose.

8. Lines 67-69: It comes across as outdated to use the term “developed” in this sentence, and in other places in the manuscript, especially since the rest of the manuscript uses the term LMIC.

Response

We replaced “developed countries” by “High income countries” and “least developed countries” by “low and middle income countries”.

9. Lines 72-74: The meaning of the sentence is not clear, does direct obstetric infections account for 11% of all maternal deaths, while in sub-Saharan Africa they constitute 12%? The next sentence also needs rephrasing, do you mean the third most common cause of maternal mortality?

Response:

We have rephrased, and it reads now:*“Globally, direct obstetric infections represent the third cause of maternal mortality. They are responsible for almost 11% of all maternal deaths worldwide; specific to sub-Saharan Africa and South Asia, these numbers rise to 12% and 14% respectively”*

10. Line 98: Maternal mortality is a ratio, not a rate, I would suggest to change to be precise.

Response: We have changed “rate” to “ratio”

11. Lines 126-127: It is not clear to the reader if Lithuania and Uruguay are considered high-income countries or were excluded for other reasons. Please clarify.

Response:

We added a statement: *“which were also classified in the group of High-Income countries as per the World Bank classification”*, to clarify

12. Lines 173-174: Did you aim to create three groups of equal size? If so, why?

Response:

Yes, we aimed to create three groups of equal size (terciles) to categorize the facilities. Because there are many items in each of the three dimensions. So we beleive the best way was to use them to assign a score to the facility base on what it has. The facilities are then ranged into 3 categories of equal size (low, medium, and high availability) because there is no benchmark to define a cut-off point.

13. Lines 184-185: It should be specified that you refer to the total number of women with SMO due to infection, not SMO overall.

Response:

Thank you! We have addressed this by adding “infection-related” to clarify. It now reads: *“We then calculated the proportion of women who had infection-related SMO among all women with infection and the rate of infection-related SMO per 1000 live births with their 95% confidence intervals (95%CI), stratified by region”*

14. Lines 196-200: If no identifiable information about individual women was entered into the system, it could not have been possible for them to request the withdrawal of their data. Please rephrase to what correct.

Response

We thank the reviewer for drawing our attention to this. The confusion may be due to the fact that we did not add that there were study logs in which we had participants’ identifiable information (along with their study participant ID). But anything that could allow someone without the log to identify a woman, was not entered into the system. It was rather kept confidential by the hospital coordinator, for him to retrieve any woman in case of claim.

15. Lines 205-206: It is common practice to name the most frequent observation first, in this case the Americas before Africa.

Response

We addressed this comment and the paragrapgh now reads: *“Most women were from the Americas (31.2%, n=788) and Africa (30.2%, n=718)”*

16. Lines 206-207: I cannot find the number 977 in Table 1, do you mean Women who had a maternal infection (n=2466)? I cannot see a separate column for women with infection with complication.

Response

We want to thank the reviewer for this comment. It allowed us to correct two things. First, we added two lines for women with complicated and less severe infections in Table 1. This is to be consistent with previous publications of the GLOSS. Secondly, we corrected the number 977 to 577 because it was an error. The total number of infections with complications is 577. This does not include the SMOs.

17. Line 240: Table 2: I would suggest to put % in italics to increase readability.

Response

Addressed. This will depend on the journal’s guidelines.

18. Lines 261-263: It is unclear to me what S5 Figure depicts as there is no legend to the figure.

Response

The information on S5 is related to that presented on Figure 1. It shows the percentage of women with SMO with their confidence intervals. We added this legend at the botton of the text as required by the journal guideline for authors.

S5 Figure: Percentage (%) of women with infection-related severe maternal outcome by region

19. Lines 283: Table 2 includes unadjusted OR, this step is not mentioned in the methods section. Please revise.

Response:

We mentioned this in the methods section and it now reads as follow: *“We computed crude, then adjusted odds ratios (aOR) with their 95%CI using a backward stepwise approach with facility-level characteristics.”*

20. Lines 292-293: It is more common, and accepted, not to report p-values among results due to its narrow interpretation, and misinterpretation. I would suggest to remove the sentence or rephrase.

Response: Thank you. We removed the p-value in the sentence.

21. Line 303: Be consistent, SMO is used in the rest of the manuscript and should be used also here.

Response: Thank you. We revised accordingly.

22. Lines 348-350: The authors should phrase this finding with great caution since the number of maternal deaths in Southeast Asia only amounted to 3 in the study period. The short duration of the study and the extremely rare outcome makes it difficult to compare, and this caution should be communicated clearly to the reader.

Response:

We thank the reviewer for this comment. While we agree with him that some caution is needed here, we want to indicate that this paragraph is about SMO, not only maternal death. So, the frequency is 21 in SEARO, not 3. But still, since this comment is relevant, we added the mention in the limitations.

23. Lines 373-377: The sentence is long and complicated, please consider revising.

Response:

We have broken the sentence into two and removed unnecessary wording. We also moved these sentences to the strengths section at the bottom of the discussion. It now reads: *“In addition, we used the Principal Component Analysis to evaluate the availability of equipment and services and the hospital's capacity. This approach allowed the computation of indexes of three dimensions of the hospital’s capacity to identify and manage maternal infection across the regions.”*

Reviewer #3:

This is a subanalysis of GLOSS study to evaluate certain regions and compare resources and outcomes and report disparities. My major concern is that the comparisons may not be valid based on the methods (see below). Descriptive info is very important and can highlight why certain regions have a greater proportion of case fatality rates and infection rates. The following is my suggested revisions to enhance the article:

1. Intro: lines 60-61 please use the WHO def of maternal sepsis rather than this layperson definition

Response: We replaced the sentence by the WHO definition of sepsis

2. lines 66-79, please consider arranging by infection on a global level, then focus on the specifics to sub-Saharan Africa and South Asia for clarity.

Consider shortening the intro as it is lengthy.

Response:

We addressed these two comments at once by removing the long paragraph on maternal mortality and bringing specific information on sub-Saharan Africa and South Asia at the end of the paragraph.

3. Methods: lines 180-189, can you please comment on how the final model was adjusted? Where all variable treated equally? Or where there adjustments based on how the income affected the resources, etc.? I would think that the lower the income, the more that might affect the capacity of the hospital to identify and treat infection.

Response

Thanks for this input. We conducted a multivariable logistic regression with a robust variance. We added all potential variables and removed those which did not improve the model. Our final model was adjusted on the following variables: income level, facility size, location, capacity to manage maternal infections, and the availability of a routine training program on infection management.

Yes, the income level affects the capacity of the hospitals. The reviewer is right; there is a correlation between income level and the capacity of hospitals to manage infections. Therefore, the model should not have contained both variables. In addressing the reviewer’s comment, we fitted the model again without the income level and corrected the aORs and their confidence intervals. We also updated the interpretation accordingly. The difference between Africa and EMRO is now statistically significant. Also note that the adjustment variables are now: facility size, location, capacity to manage maternal infections, and the availability of a routine training program on infection management. We would like to thank them for raising this.

4. Table 1: Please consider just spelling out the abbreviations. It's hard to follow even with the key at the bottom.

Response: We spelled out all the abbreviations in the table

5. Table 2. There is a lot of variability in the number of facilities in each region. How was this managed?

Response

Yes, there is variability because for some variables, like “Staff availability”, the frequencies are in the category “yes.” So, we added this mention in the table to clarify. Otherwise, we did not replace or impute the missing values because there were not many, and they were not in the model.

6. Table 3: These comparisons are not particularly helpful since the regions are not equal and I am not sure the model adequately adjusts for the differences.

Response:

These comparisons are exploratory because we cannot ascertain whether all the regions’ factors needed to fit this model were available. But we tried with the data we had to fit the best model we could, using *AIC, BIC,* and the *linktest* to select the final model. After this, though we would say that future studies can improve and get a better estimate of the OR, the direction of the differences is somehow consistent. This caution has also been mentioned in the discussion.

7. Discussion: this should be shortened significantly.

Response:

We thank the reviewer. We have shortened the discussion section by removing information that was not mandatory for the clarity and flow of the paper. Please see the manuscript with tracked changes.
